# Using a Community-Based Participatory Mixed Methods Research Approach to Develop, Evaluate, and Refine a Nutrition Intervention to Replace Sugary Drinks with Filtered Tap Water among Predominantly Central-American Immigrant Families with Infants and Toddlers: The Water Up @Home Pilot Evaluation Study

**DOI:** 10.3390/nu13092942

**Published:** 2021-08-25

**Authors:** Shannon McCarley, Mairyn López-Ríos, Rosalina Burgos Gil, Monique Mitchell Turner, Sean D. Cleary, Mark Edberg, Uriyoán Colón-Ramos

**Affiliations:** 1 Global Health Department, Milken Institute School of Public Health, The George Washington University (GWU MISPH), Washington, DC 20052, USA; slmccarley@gwmail.gwu.edu; 2 Insight Policy Research, Arlington, VA 22209, USA; Mlopezrios@insightpolicyresearch.com; 3 Senior Director of Early Childhood Education Programs, CentroNía, Washington, DC 20009, USA; rburgos@centronia.org; 4 Department of Communication, Michigan State University, East Lansing, MI 48824, USA; mmturner@msu.edu; 5 Department of Epidemiology, GWU MISPH, Washington, DC 20052, USA; sdcleary@gwu.edu; 6 Department of Prevention and Community Health, GWU MISPH, Washington, DC 20052, USA; medberg@gwu.edu

**Keywords:** community-based participatory mixed methods, Latinos, infants and toddlers, sugar-sweetened beverages, tap water

## Abstract

Descriptions of the implementation of community-based participatory mixed-methods research (CBPMMR) in all phases of the engagement approach are limited. This manuscript describes the explicit integration of mixed-methods in four stages of CBPR: (1) connecting and diagnosing, (2) prescribing-implementing, (3) evaluating, and (4) disseminating and refining an intervention that aimed to motivate Latino parents (predominantly Central American in the US) of infants and toddlers to replace sugary drinks with filtered tap water. CBPMMR allowed for co-learning that led to the identification of preliminary behavioral outcomes, insights into potential mechanisms of behavior change, and revisions to the intervention design, implementation and evaluation.

## 1. Introduction

As dietary and health disparities continue to persist in the United States (US), community-based participatory research (CBPR) approaches have increasingly received attention as a potentially effective way to leverage research to improve health in low-income marginalized communities [1,2,3]. Approaching research from a CBPR lens, members from academia partner with community entities to engage in co-learning in order to better identify and address community priorities and needs [4,5]. Therefore, CBPR approaches are expected to: develop interventions that are more relevant to the problems that the community wants to solve; generate better-informed hypotheses with scientifically valid approaches; incorporate community norms and values into intervention strategies; increase the accuracy and cultural sensitivity in the interpretation of findings; and increase the potential for translating of evidence-based research into sustainable community changes [6]. In addition, CBPR approaches have been shown to improve recruitment capacity, increase community member capacity, increase the quality of program implementation, as well as outputs and outcomes, increase sustainability of project, and lead to system changes [7].

CBPR approaches can benefit from employing both quantitative and qualitative methods within a single sustained scientific inquiry to facilitate co-learning and strengthen discussions that can inform solutions towards health justice [5,8]. The systematic integration of quantitative and qualitative data during the various aspects of the investigation processes within a single line of inquiry is part of mixed-methods research [9]. Mixed methods research allows for flexibility and iteration to provide information-rich data that can capture the lived realities of the communities as these emerge [8]. Nonetheless, the use of mixed methods in CBPR research is seldom described beyond intervention design/planning and implementation [10]. These descriptions are important for future work that seeks to abate dietary and health disparities.

This manuscript describes in detail a multistage mixed methods study that followed a CBPR approach in problem identification, study development, evaluation, and refinement of an intervention to address a dietary disparity: high sugar-sweetened beverage consumption (SSB) and low consumption of plain water in a diverse Latino community. Latinos are among the demographic groups in the United States with the lowest water consumption and highest SSB intake [11,12] and are less likely to give tap water to their children because they do not trust the local water or do not like its taste [13,14,15,16]. To date, there have been few interventions that aim to motivate low-income minority families, especially those with very young children, to decrease SSB consumption and to offer an alternative, such as plain drinking water [17]. These interventions face the unique challenge of mistrust of tap water, which could be an important barrier to decreasing SSB consumption [18]. The description of the integration of mixed methods in all phases of CBPR can offer important insights into how to approach diverse communities to address dietary disparities in future work.

## 2. Materials and Methods

**Figure 1:**
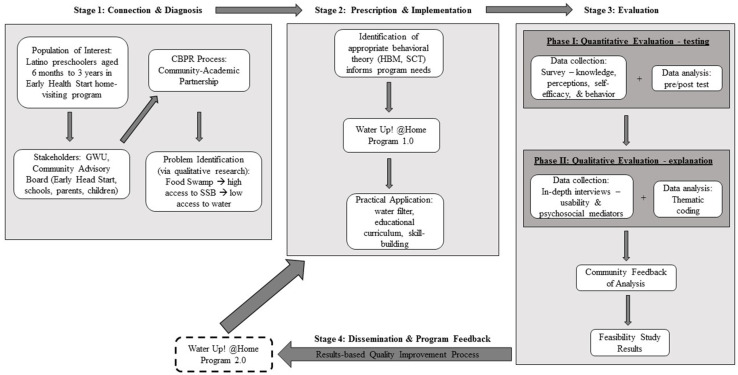
Diagram of Community-Based Participatory Research (CBPR) Multistage Mixed Methods Study. Iterative CBPR process and parallel use of quantitative and qualitative methods to diagnose the problem (Stage 1); Theoretical framework developed, and program implemented (Stage 2); Two-phase explanatory sequential evaluation (Stage 3); Dissemination of mixed methods results, program feedback (qualitative methods), and revision using meta-inference from mixed methods (Stage 4). GWU: George Washington University; SSB: Sugar-sweetened beverages; HBM: Health Belief Model; SCT: Social Cognitive Theory.

Throughout this section, we use the work of Cresswell and Plano Clark’s (2011) and Stoecker’s (2016) to guide descriptions of the integration of mixed methods into CBPR through four stages: (1) connecting with the community and diagnosing the scientific line of inquiry (eventually defined as high consumption of SSB and limited access to an alternative such as tap water), (2) prescribing-implementing (the focus hereon is on one particular intervention addressing the parents of infants and toddlers in the home visiting program of early child care centers), (3) evaluating the intervention, and (4) disseminating findings and refining the intervention (Figure 1).

### 2.1. Stage 1—Connecting and Diagnosing

The first stage describes the community-academic partnership and the formative work conducted to diagnose the public health problem that was relevant to the community. During this stage, the use of mixed methods was exploratory (qualitative, followed by quantitative), and mixed-methods findings were integrated during the frequent (monthly) discussions with the community advisory board to define the scientific line of inquiry.

#### 2.1.1. Connecting with the Community

The community-academic partnership originated 12-years prior to the nutrition work presented here. The partnership originally focused on addressing the mental wellbeing of Latino immigrant youth, especially undocumented youth. In 2014, the community was characterized by being 80% Hispanic, 68% foreign-born (predominantly from Central American countries), and low-income (1 in 4 children lives in poverty) [19]. The partnership was guided by a community advisory board (described in detail elsewhere: [20]). In brief, the community advisory board consisted of active participation from 8–10 members during each monthly meeting; the board composition sought representation of community residents (*n* = 2–5), representatives from non-governmental organizations that serve this community (educational, legal, early care, health and wrap-around services (*n* = 4–6), school teachers in their personal capacity (*n* = 2), and researchers from the academic institution (*n* = 4–6). Some, but not all, of the researchers were also residents of this community and were Latino and/or Spanish-speakers. All community advisory board meetings were facilitated in both Spanish and English by a bilingual Latino community organizer who had more than 40 years of experience in organizing, including formal community organizing training, conducting and facilitating plenary discussions, as well as qualitative and quantitative data collection for private and public entities. This organizer was hired by the academic partner. The bilingual agendas for all meetings and discussions were prepared by the academic partner and open for feedback from community members. All meetings allowed time and space for updates from community advisory board members, as well as concerns and additional points for discussion. Minutes were provided to the community advisory board members after each meeting in both Spanish and English.

Under the original focus of work, the academic partner led a series of exploratory Photovoice activities with community youth as part of a positive youth development intervention [21]. One of the topics that emerged was a concern about lack of access to nutritious foods in the community. To further refine and narrow the concern to an actionable research question, the academic partner sought additional grant support from a federal agency (CDC Racial and Ethnic Approaches to Community Health). The academic partner led the governance of the cooperative agreement including the following activities: approval to conduct research, oversight and regulation of research ethics, leadership of research design and implementation, recruitment of research staff and participants, oversight of partnerships and resources, and oversight and management of data and dissemination.

#### 2.1.2. Exploratory Mixed Methods for Diagnosis

Grounded and guided by the long-standing community-academic partnership, exploratory mixed methods were employed to formulate an actionable research question. When collecting data from Spanish-speaking participants, data collection instruments were designed in Spanish by a member of the academic partner who was a native Spanish speaker. All data collection instruments were subsequently discussed with the community advisory board, tested for comprehension with members of the community, and eventually translated to English for IRB purposes and for discussion among non-Spanish speakers of the research team. All data collection, analysis and dissemination protocols were reviewed and approved by the academic partners’ office for Human Research (protocol #s 101648; 051503; 011517).

***Photovoice interviews and a Culminating Workshop with mothers*:** Research protocol and findings of this Photovoice activity have been published elsewhere [22]. In brief, the academic partner (native Spanish-speaker) designed two Photovoice interviews and a group culminating workshop to explore which barriers and facilitators mothers in the community faced in feeding their children what they wanted to feed them. Self-identified Latino mothers (*n* = 15) who had children < 10 years old were recruited by the community organizer. The culminating workshop was led by the academic partner and aimed to identify concrete strategies to address these barriers. Members of the community advisory board were invited to attend the workshop as listeners and were allowed to ask clarifying questions. All activities were conducted in Spanish, audio-recorded and transcribed verbatim. Transcripts were provided to the bilingual members of the academic partner for deductive thematic analysis.

***Integration of results and discussion with the community advisory board:*** The main findings from this Photovoice activity were then summarized in Spanish and translated to English by the academic partner and shared with the community advisory board. During the discussion, one topic in particular resonated with the community advisory board: mothers wanted to provide water for their children because they considered it to be healthier than sugar-sweetened beverages (SSB), but they did not trust the tap water. Members of the community advisory board who were residents of the community shared the same perception about the tap water. Some had also witnessed the high SSB consumption among residents in this community, including very young children, and were concerned about potential health risks associated with this consumption, but did not have the hard data to justify this concern or tools to collect such data.

***Literature review and discussion with the community advisory board:*** Upon these discussions, the academic partner reviewed the existing scientific and grey literature on tap water and SSB consumption among Latinos nationwide. The literature was summarized and presented to the community advisory board, emphasizing that SSB was a risk factor for obesity and diabetes, and that they were commonly consumed and heavily marketed to Latinos [23,24,25]. Research also documented that Latinos consumed significantly more bottled water than non-Hispanic Whites, sometimes spending up to 16% of their annual income on bottled water, and that they were less likely to trust their local tap water [14,15,16]. The board discussed the potential implications of a cross-sectional association between perception of tap water safety and greater SSB consumption in one study where Latino youth who perceived their school tap water to be unsafe had 2.9 times greater odds of consuming SSBs daily compared with those who were neutral or agreed that the water was safe [18].

The literature review and discussion ensued the following concerns among members of the community advisory board: (1) the environmental impact of plastic water bottles was perceived as problematic by some members who were working with school principals to certify schools as environmentally-friendly [26]. (2) Members of the community advisory board had seen or experienced themselves severe dental caries problems, ensuing a discussion during meetings about potential prevention of dental caries if fluoridated tap water was consumed instead of SSB and/or bottled water. The academic partner contributed findings from the published literature to document that in the United States, Latinos and their children were disproportionately burdened by dental caries [16,27,28]. (3) Residents in the community were concerned about the cleanliness and safety of tap water in the county. Members of the water sanitation committee of the county were invited to share how tap water was treated and fluoridated locally. (4) Members who worked in schools and early child care centers were concerned with the high availability and promotion of SSB in the community. The academic partner led the community advisory board on a guided walk to all food retail in the community, where they recorded the type of food retail (restaurant, grocery store, corner markets), in-store promotion for different beverages, and whether there was access to drinking water points (e.g., bulk bottled water, coolers, filtered water stations, kitchen sinks, fountains, cups available for water). The observation protocol was designed de novo by the academic partner based on a review of existing literature [29,30]. Findings of the 15 markets and 44 prepared food sources observed were then discussed as follows: the majority of the markets and prepared food sources catered to the Latino community, promoted sodas and ethnic sodas, but not all offered bottled water, and the larger grocery store catering to Latinos offered a free 3-L of soda when customers spent more than $50 on groceries. None of the Latino-catering independent restaurants offered free drinking water upon seating, would hesitate or refuse to serve free drinking water if asked by customers, and promoted sodas and home-made sugary drinks such as *horchata or aqua de jamaica.*

***Definition of the area for further scientific inquiry:*** These initial discussions pinpointed the community advisory board’s interest in better understanding beverage consumption behaviors and their determinants, especially perceptions of tap water safety and SSB and water consumption. The board decided to focus the first formative work data collection to occur in schools where SSB or water are typically promoted and consumed among school-aged children. It is important to note the contextual factors that may have influenced these decisions of work focus at time, including the First Lady’s campaign to drink water instead of SSB and the promotion of the 2010 national recommendations that drinking water should be freely available to children where they eat [31,32].

***Formative data collection:*** Formative data were thus collected in these settings, starting with qualitative data in schools (10 in-depth interviews in Spanish or English, as preferred by the interviewees who were school teachers and principals in five elementary schools of the community) and with school-aged youth (10 focus group discussions in Spanish [33]). Interview guides and focus group discussion guides were developed in Spanish, and translated to English by members of the academic partner who were proficient in both languages. These were discussed with the community advisory board for further feedback prior to submission to the IRB. The guides sought to describe perceptions of tap water for drinking, behaviors related to water (bottled, tap) and SSB consumption at home and at school, and sought to identify potential intervention strategies. The methods of the focus group discussion have been described in detail elsewhere [33].

Data were collected by a formally-organized group that has over 20 years of experience in training Latino residents and students to collect quantitative and qualitative data (this group is called the Rivera Group and herein referred to as the RG). Members of the RG are all Latino, native Spanish speakers, predominantly immigrants from Central American countries and residents in the community. The RG group, which has worked with the academic partner in previous projects, was hired and trained by the academic partner to recruit and consent participants, collect and transcribe the qualitative data. Data collection was conducted in Spanish or English, depending on the preference of the participants. Transcripts were provided to the academic partner in the original language that data in which they were collected. The academic partner analyzed transcripts for thematic organization according to three broad categories: beverage consumption behaviors, perceptions of tap water, facilitating factors and barriers to consuming water in schools and at home, and potential intervention strategies.

***Integration of qualitative results and discussion with the community advisory board:*** Qualitative findings were summarized by the academic partner in Spanish, translated to English, and presented in both languages to the community advisory board. The findings from these interviews suggested that school staff drank only bottled SSB purchased from vending machines or bottled water brought in from home. They did not use the water fountains at school because they did not perceive the water to be clean. They also reported that students were not allowed to bring reusable water bottles to school, and that schools were not able to provide access to free drinking water during lunch time. Findings from the FGD were in agreement: youth described that water was not available at schools and that they preferred to drink flavored sweet milk during lunch time at school. Youth did not drink tap water at home either. Upon these findings, the community advisory board was interested in understanding the level to which the community was ready to promote drinking water, primarily from tap.

***Community Readiness Interviews:*** The academic partner trained six members of the community advisory board to conduct community readiness interviews using the protocol and script outlined in [34], which consists of 36 open-ended questions to assess the degree to which a community is prepared or ready to implement change related to a specific issue. The community advisory board met to discuss which initial set of key informants would be selected for the interviews, based on their availability and knowledge of the community. A snowballing technique was used to select other key respondents in the sectors of health care clinics, non-governmental organizations working on health, and county government representatives working on health. A total of nine interviews were conducted by community advisory board members. The interview length varied widely (10–60 min). All interviews were audio-recorded when allowed; transcripts and notes were provided to the academic partner, who analyzed those following individual scores for each one of the dimensions as described in [34]. Final scores indicated the community was in the stage of ‘no awareness’ in their efforts or resources linked to promotion of drinking tap water. Barriers to this promotion included cultural perceptions about drinking tap water, perceptions that fruit-flavored SSB were healthy, and competing priorities, such as providing ‘calories’ for the family.

***Intercept Surveys:*** After discussions of qualitative findings, the community advisory board aimed to understand if the perceptions about tap water safety and palatability emerged from country of origin, given their perception that tap water is not potable in some regions of Central America. The academic designed *de novo* a rapid assessment survey to expand this discussion. The 21-item survey was developed in Spanish to ask about perceptions of tap water in country of origin and in the United States. The RG collected 130 surveys in Spanish from a convenience sample at various community events. Findings were compiled and summarized by the academic partner, translated into English, and presented in both languages to the community advisory board. Findings suggested that 52% of respondents drank tap water in their countries of origin because they considered it to be convenient, they liked its taste, and they perceived that it was safe and economical. However, once in the United States, only 9% of these same respondents drank tap water, mostly because of concerns about its safety, taste, and because bottled water was more convenient to just grab when they were on the go.

***Focus of Actionable Research Question:*** The eventual diagnoses of a scientific line of inquiry (high SSB consumption and low water consumption, perhaps because of perceived limited access to safe, palatable tap water) arose as a product of these discussions and led to identification of strategies for action in various community settings (schools, restaurants, early child care settings, and the community at large). The academic partner led the discussion to define research questions, study designs and data collection tools for each of these strategies that aimed to change access to tap water to increase water consumption and decrease SSB consumption in the various settings.

The rest of this manuscript focuses only on the interventions designed and evaluated for the early child care setting because the strategies used in this organization were systematically evaluated using a two-phase explanatory sequential mixed methods design and subsequently disseminated and refined with community input, thereby illustrating all the phases of CBPMMR illustrated in Figure 1.

### 2.2. Stage 2—Prescribing-Implementing (Design of Intervention)

The leadership of the early child care organization was an active member of the community advisory board. The organization participates in the federal Early Head Start program and they already had implemented internal policies in all of their centers in order to offer only plain water or plain milk to the children in their centers. However, they were now interested in reaching the homes of their participating families through their existing Early Head Start home-visiting program. The following activities describe the co-learning process that was facilitated by the CBPMMR approach.

#### 2.2.1. Co-Learning to Develop Theoretical Framework

Drawing from the formative work that described the mistrust in tap water safety and cleanliness and informed by the crisis that occurred in Flint, Michigan at the time of the formative data collection [35], the academic partner first researched various models that could facilitate discussion about potential intervention given the emerging context and concerns of the community. The community advisory board narrowed down to the Integrative Environmental Health Model, which has been previously evaluated in the assessment of perceptions about tap water quality among minority children [36] and aligned with themes used to illustrate the causes of drinking water disparities in the United States [37]. This model draws from the Health Belief Model [38], which assumes that health behaviors are determined by the perception of susceptibility to a problem, the seriousness of the problem (severity) and which prevention activities are effective and not overly costly, as well as exposure to a cue to take action. With the community advisory board, the following narrative was developed to explain the use of the model as applied to the line of scientific inquiry in this community: children are excessively exposed to SSB and need clean, fluoridated water for appropriate growth; living in a disadvantaged community with potential infrastructure disparities that can affect water quality (vulnerability domain) can lead to exposure (or perceived exposure) to dirt and color cloudiness of the tap water (physiological domain), and reinforce negative personal thinking and social knowledge about tap water (epistemiological domain). This can result in greater consumption of bottled water and other bottled beverages, such as SSB (health protection domain). Arguing that all of the domains must be addressed simultaneously in order to achieve health protection action, this model facilitated identification of intervention strategies for each domain.

#### 2.2.2. Addressing the Theoretical Constructs of Knowledge, Susceptibility, Severity, Costs and Benefits in the Vulnerability, Epistemology, Perceived Physiological, and Health Protection Domains via a Curriculum

A first draft of a bilingual (English and Spanish developed in parallel) curriculum was developed by members of the academic partner. The curriculum aimed to increase knowledge and perceptions of susceptibility, severity, costs and benefits regarding the four domains. The vulnerability domain was addressed with information regarding susceptibility and severity of diabetes and obesity among Latinos. Vulnerability and perceived physiological domains were addressed with information regarding safety and cleanliness of tap water in the US, including prior experiences with tap water consumption in country of origin and in US, and perceptions of safety and cleanliness processes in the US. Health protection domain was addressed with information about health benefits of tap water (fluoridation) vs. bottled water; health benefits of water vs. health costs of sugary drinks; recipes and tips to improve the taste of tap water (cues to action). Cost/convenience of filtered tap water vs. bottled water and sugary drinks; tips to drink tap water on the go, at social and family events, places (food retail and other community areas) where residents can obtain free bottle refills with drinking water in the community to address the epistemological domain.

Bilingual drafts of the curriculum were presented to the community advisory board for active feedback and were iteratively revised during a series of meetings with the leadership of the early child care organization to address their concern about high juice consumption among infants and toddlers, and the challenges that parents may face in differentiating between fruit-flavored SSB and 100% fruit juice. Additional information about how tap water is cleaned and fluoridated to protect against dental caries; information about the sugar content of 100% fruit juices; and tips to dilute fruit-flavored SSB and 100% fruit juices were then incorporated into the curriculum.

A graphic designer was hired by the academic partner to visualize data regarding the topics above in trifold prototypes. The trifold prototypes were presented to the community advisory board and tested for comprehension and acceptability in two group discussions with Latino mothers (*n* = 8; *n* = 12), and via in-depth cognitive interviews (*n* = 20) with mothers (70% had <4th grade education). These activities were led by the academic partner with self-selected participation from community advisory board members. The brochures were subsequently revised by the graphic designer and academic team to address language (expressions more appropriate for Central America) and visuals (inclusion of photos that were more representative of Central American and indigenous ancestry, vs. stock photos of Latinos that present more European descent).

#### 2.2.3. Addressing the Physiological Domain: Real and Perceived Physical Barriers to Drink Tap Water Instead of SSB

The formative work had made it evident that community members were concerned about tap water safety and palatability. The research team could not guarantee that the tap water accessed at home would not be contaminated. The use of a water filter had emerged during community advisory board meetings as a potential solution to this concern. The academic partner researched the various options to address the potential of unsafe tap water at home, and presented them to the community advisory board. The community advisory board decided on a National Sanitation Foundation-certified water filter pitcher (~99% removal of lead and impurities, not fluoride, with an approximate 3-month lifespan) and a reusable water bottle to facilitate access to filtered tap water consumption when parents left the home.

#### 2.2.4. Increasing Skills and Self-Efficacy

The leadership of the early child care organization met additional times to jointly develop a series of hands-on activities for each curriculum lesson designed to improve parents’ skills and confidence in replacing SSB with filtered tap water. These included: collecting families’ SSB sales receipts weekly to make parents aware of how much they were spending on SSB (compared to the cost of filtered tap water) and thereby aim to increase their skills in budgeting and confidence in making the switch; increasing parents’ skills in assessing sugar content in beverages that they commonly consumed; and increasing parents’ media literacy by becoming aware of promotions of SSB in their neighborhood by taking pictures of these. In addition, parents were asked to take pictures and videos of themselves explaining to their family members the key messages from various lessons, using the reusable water bottle, asking for water instead of SSB at a restaurant, and drinking filtered tap water and offering it to their children, family, and friends. These activities were intended to position participating parents as ‘models’ of the targeted behaviors.

#### 2.2.5. Implementation Design

The intervention strategies (informational curriculum, self-efficacy activities, and water filter provision) were designed to be delivered during the existing Early Head Start home visiting program of the early child care organization. This organization already conducted weekly home visits for 90 min with trained home visitors employed by the child care organization. During the study period, the home visitors integrated the curriculum and other intervention strategies into their weekly lessons for 12 consecutive weeks. The lessons were designed to be conducted in 40–45 min of the 90 min sessions.

### 2.3. Stage 3—Evaluation of Implementation and Impact

#### 2.3.1. Methods of Stage 3

A multi-phase sequential mixed-methods explanatory design was selected to evaluate the intervention strategy and deepen our understanding of its implementation and potential for behavioral impact [9]. The academic partner took the lead on designing the evaluation components and training the RG group and academic research assistants to collect and analyze data. The early child care organization took the lead on the recruitment and implementation of the program following inclusion criteria: all Latino adult (>18 years of age) parents who were enrolled in the home-visiting program of the community partner were invited to participate in the study.

***Phase 1: Quantitative Data Collection***: Feasibility of the program implementation was meant to be assessed via short-answer evaluation sheets designed by the academic partner to be completed weekly by the home visitors (one per family). The original evaluation sheets included short-answer questions which asked about the duration of the lesson, home visitors’ assessment of how parents understood the lesson, home visitors’ impressions about the adequacy of the content, and potential barriers or challenges they faced during the lesson delivery. After consultation with the early child care organization, these were revised to include checklists to ease completion.

To assess potential for behavior change and theoretical underpinnings, baseline and follow-up surveys were developed by the academic partner using the following variables:

*Parents’ knowledge* was measured using seven close-ended questions developed *de novo* to assess knowledge about: (1) where tap water comes from; (2) how SSBs are marketed to Latinos and how to calculate the quantity of sugar in a SSB from the product label; and (3) the health costs of drinking SSB and the health benefits of drinking water. Participants could score up to 11 correct points.

*Parents’ skills and self-efficacy* in how they responded to cues to ‘action’ were assessed using 19 close-ended questions developed *de novo* to ask about capability/confidence to actions to: (1) replace SSB with water; (2) replace bottled water with tap water and with filtered tap water (asked separately); (3) increase water consumption; (4) act as models for their children, family members and during social gatherings; and (5) decrease SSB consumption for themselves and for their children. Participants could score up to 57 points.

*Parents’ tap and bottled water consumption and preference* including perceptions and beliefs about water in the United States, were collected using items adapted from a previous questionnaire [15].

*Sociodemographic information* (age, sex, country of birth, years in the United States) of the participating parent were collected at baseline.

*Beverage intake* among parents was assessed using an established self-reported survey about habitual consumption of 19 beverage categories in the past month for adults [39] and for preschoolers [40]. Both questionnaires have reported acceptable reliability and validity among adults [39] and among Hispanic preschoolers [40].

The proposed survey was shared with the early child care organization for advice on constructs, question wording, and additional topics. Prior to survey administration, it was tested for comprehension in English and Spanish among parents who were residents of this community. From this process, the following adaptations: (a) asked separately about bottled and tap water for children; (b) added examples of common or culturally popular drinks to the list; (c) added questions about energy drinks and sweet coffee for children.

All survey data were collected by the RG group in the language of preference of the participant (English or Spanish). Participants received a gift card ($25) for each survey. The early child care organization attended and co-led training sessions, with a special focus on the consent protocols.

**Phase 1: Quantitative Data Analysis:** Implementation Data Analysis: The home visitors turned in to the academic partner weekly evaluation sheets (in paper) for each family. Data were manually entered into Excel Sheets to calculate the average duration of each lesson and to capture any other comment about whether parents understood the lessons.

*Survey Data Analysis:* All quantitative data were collected in paper surveys, entered into Excel and exported to a statistical analysis package (STATA version 15) for analyses by the academic partner. A research assistant trained in quantitative data analysis checked all outcome variables for normality; interquartile ranges and median values were used if variables were not normally distributed. SSB consumption was defined as the sum of oz/day consumption of regular soft drinks, fruit-flavored SSB, sweet coffee/tea, flavored milk with added sugar, sweet caffeinated drinks, and energy drinks as in previous analyses [41,42,43]. Missing data was excluded from analyses. A t-test for continuous variables and a Chi-Square or Fisher’s Exact test as appropriate for dichotomous categorical variables were used to assess any differences between baseline and follow-up values.

**Phase 2: Qualitative Data Collection:***In-depth interviews with participants*: The academic partner developed a first draft for a semi-structured in-depth interview guide with the intention to provide depth and context to the survey findings. The guide was discussed with early child care organization and subsequently refined to elicit descriptions about participants’ impressions of the intervention strategies and to have participants describe how they may have chosen beverages through and after the intervention (*i.e., ‘What barriers do/did you face as you choose/chose what beverages you and your children drink at home*?’).

Interviews were conducted by a Latino-descent, bilingual (Spanish/English) research assistant. All parents who participated in the intervention and follow-up survey were re-contacted and invited to participate in the interviews. Interviews were conducted in Spanish, lasted 20–40 min, were audio-recorded, and transcribed verbatim. No additional demographic data were collected at this time. Participants received a $20 gift card) for their participation.

*Group Interviews with Home Visitors:* The academic partner prepared and conducted an interview guide to elicit group feedback from the home visitors into the implementation and its perceived effectiveness. This was done in two groups (*n* = 5 each, total of 10 home visitors) in Spanish. Home visitors were asked to rank all lessons according to two criteria: (1) their own preferences regarding how easy each lesson was for them to implement, and (2) what they perceived were their families’ preferences. They were also asked to share which aspects of the curriculum they would change, why, and how. Home visitors received a $25 gift card for their participation.

***Phase 2: Qualitative Data Analysis:*** All transcripts were entered into ATLAS.ti. For the in-depth interviews, a bilingual research assistant trained in qualitative research methods by the academic partner completed an initial deductive coding based on the domains of the theoretical framework. In further conversations between the early child care organization and the academic partner, the domain of ‘modeling behaviors’ was added to assess if parents were engaging in desired behavior to be a ‘role model’ for themselves and their children. During the test-code, several initial themes were identified based on response patterns, and the coding strategy was revised. The codebook was revised continually as additional themes emerged inductively, following best practices for inductive-deductive qualitative research [44,45]. These code additions included: change in knowledge of the intervention topic, sustainability of the intervention, including sub-codes for comprehension of the information, program impressions, and program suggestions. A second, independent coder reviewed the coding strategy, further refinements were made in a collaborative fashion, and the final coding strategy was applied independently to recode all interviews and compare reliability.

For the group interviews with home visitors, all discussions were audio-recorded, transcribed verbatim and summarized by the academic partner into the following categories: (1) what worked; (2) what didn’t work as well; (3) recommendations.

***Integration of Phases 1 & 2 mixed methods in the analysis***: Quantitative findings were summarized and presented to the early child care organization by outcome, each followed by a summary of the qualitative themes and potential illustrative quotes. A discussion ensued to provide clarification, interpretation, and depth to understand the quantitative results in light of the qualitative explanations. The authoring team of this manuscript, composed of academic partners and the leadership of the early child care organization, worked together on meta-inferences from the integrated results and presentation of the findings [46].

#### 2.3.2. Results of Stage 3 Intervention Evaluation

***Implementation/Feasibility Results:*** The average duration of each lesson was 31 min; 95% of the home visitors thought the lessons were adequate and home visitors reported that parents understood the lessons in 99% of the weekly evaluations.

*What worked:* During the group interview, home visitors expressed that time was adequate to implement each lesson; that the parents learned from the lessons and applied the information to reduce the amount of sugar in their beverages, especially if prepared at home. Among the aspects that they perceived worked, they mentioned: the information included in the intervention was impactful (sugar content of SSB in light of added-sugar upper limit recommendations); provided useful cues to action (reusable water bottle; water filter); made parents aware of pervasive availability and marketing of SSB vs. water in food retail. The engagement activities for the parents (preparing fruit-infused water; measuring the amount of sugar in drinks they consumed in light of recommendations) also received positive feedback.

***What didn’t work as well****:* Home visitors would have preferred flexibility in the delivery of the curriculum. They often shortened or combined lessons to take into account unforeseen scheduling conflicts. Some of the activities designed to increase parents’ self-efficacy (i.e., recording videos of or taking photos of themselves to model behaviors; keeping sales receipts) were too time-consuming and parents did not see the benefit of completing them. Some of the families needed replacement filter cartridges before the program implementation ended. One of the home visitors lost the reusable water bottles and therefore, never delivered them to the participating families. Home visitors were often required to simultaneously deliver the Water Up! @Home intervention and the normal Early Head Start curriculum during the same visit because they needed to report on child development milestone indicators during this time. Delivering two curricula simultaneously made the visit cumbersome and unworkable. The home visitors described how the parent-centered lessons and activities left the infant or toddler unoccupied and unattended during the visit.

***Recommendations for future implementation:*** Parent modeling of behaviors were deemed as a component that should be emphasized. Home visitors suggested that including more child-centered activities aligned better with the child development curricula that the early child care organization is required to deliver. The following additional recommendations were made: flexibility in the delivery of lessons; elimination of the activities that were too cumbersome to complete; inclusion of activities that could foster parent-child interactions.

#### 2.3.3. Mixed Methods Results for Behavior Change and Theoretical Underpinnings

*Description of Participants (*Table 1*)*: Fifty-one Latino parents who were enrolled in the early child care organization home visiting program and who did not currently have water filtration systems at home were recruited into the Water Up! @Home pilot evaluation study and completed the baseline survey. Of those, nine parents moved to a different program before starting the intervention; 39 parents completed the intervention (attrition rate: 93%), and 36 completed the follow-up survey (14% loss to follow-up).

Almost all participating parents (94.28%) were female, aged 26–40 years (78%), with <12 years of schooling (78%); 97% were born in Central America and 72% earned less than $20,000 a year. The mean age for children was 29.5 months. Additionally, 89% reported that they consumed bottled water, 6% drank tap water and 42% spent >$25/week on bottled water. When asked about water consumption in their countries of origin, 46% reported that they consumed tap water in their country of birth and only 11% consumed bottled water. Eleven Water Up! @Home participants accepted to be interviewed after completion of the intervention.

*Outcomes and Potential Mechanisms (*Table 2 and Table 3*)*. Quantitative findings are presented first, followed by the explanatory qualitative findings and meta-inference from mixed-methods results.

##### Theme 1: Knowledge Gains after Intervention

Parental knowledge scores increased significantly 1.07 points (*p* < 0.05) after the intervention. The interviews provide unprompted, spontaneous descriptions about increased knowledge related to health risks associated with SSB and health benefits of drinking water. These themes reached total consensus across the qualitative interviews (Table 2, quote 1.1). The majority of interviewees expressed that after the intervention they understood that tap water was cleaned and treated in the United States (Table 2, quote 1.2); they knew how to calculate the amount of sugar in a beverage by converting grams to teaspoons, and they knew the recommended maximum intake of sugar for men, women, and children (Table 2, quote 1.3). There were a few specific and explicit mentions of diabetes, obesity, and dental caries as a result of SSB consumption (Table 2, quote 1.4). Therefore, a meta-inference from the quantitative and qualitative findings is that the curriculum lessons provided the participants with new knowledge about key concepts regarding risks associated with consuming SSB and benefits of drinking water, specifically, tap water, and that they felt confident in describing this knowledge, unprompted, to the interviewer.

##### Theme 2: Perceptions of Susceptibility, Severity, Costs and Benefits

The surveys were not designed to ask about perceived susceptibility, severity, costs and benefits of drinking SSB vs. water. Nonetheless, these perceptions emerged spontaneously among all participants as they described what they learned (knowledge). In that sense, themes 1 and 2 were linked by the participants themselves. Specifically, the majority of participants used language such as “*risk of disease*”, “*vulnerable* [to disease]”, and “*sugar can do harm*” to describe their changed perceptions in their susceptibility to risks associated with SSB consumption and costs (harm) of SSB consumption. Conversely, water was described as “*healthier*” and “*more natural*”, compared to other beverages, and perceived as a benefit to one’s health, but Latinos were seen as more susceptible to limited water consumption (Table 2, quotes 2.1 and 2.2).

##### Theme 3: Perceived Physical Barriers to Drinking Filtered Tap Water

The quantitative survey assessed parents’ pre- and post-intervention perceptions about drinking tap and bottled water at home and giving it to their children. Although there were differences in baseline and follow-up that aligned with the objectives of the intervention, none of these reached statistical significance with the small sample size (*p* > 0.05). In addition, 81% of participants liked or strongly liked the taste of water before the intervention, and this increased to 97% after the intervention, although at the end of the intervention 58% of participants still reported that they did not provide tap water to their children because it made them sick.

The qualitative results also suggest that participants were still concerned about the quality of tap water, but the water filter dissipated some of those concerns (Table 3, Quote 3.1–3.2). All but one participant stated that after the intervention, they felt comfortable drinking filtered tap water in their home. As an overall theme, recognition of barriers to accessing and/or consuming water reached total consensus (Table 2, Quote 3.3). Participants also recognized that the intervention reduced perceived barriers to access palatable water (Table 2, Quote 3.4). There was less consensus (only 46% of participants) about the effectiveness of the reusable water bottle to increase access to filtered tap water outside the home. However, since one of the early child care organization sites never distributed the reusable water bottles to the families, this lack of consensus is difficult to interpret.

*Meta-inference from mixed methods results:* Taken together, the survey findings suggest that more parents reported consuming and providing filtered tap water to their children, although there were persistent concerns about the unfiltered tap water. The qualitative findings offer a plausible explanation: the use of the water filters seem to have mitigated some of the concerns about tap water safety and suggest that using the filter, parents were able to reduce consumption of bottled water and increase consumption of tap water for their children. These mixed methods data also contribute to identify a future area of investigation: how may the curriculum (compared to providing a water filter alone) address these concerns and be able to increase likelihood of behavior change?

##### Theme 4: Skills and Self-Efficacy

The mean score for self-efficacy increased significantly (*p* < 0.05) by 2.64 points after the intervention. Table 2, Quotes 4.1–4.2 suggest that participants put knowledge into practice by participating in the activities designed to increase their skills and confidence. All interviewees described that they learned skills to prepare fruit-infused water, assess the sugar content of a beverage, make conversions from grams of sugar in the nutrition facts label to teaspoons of sugar, and use the label to decide about which beverages to purchase or how much of the sugary beverage to serve to their children. Similarly, feeling confident to take actions to increase their own water intake, such as drinking fruit infusions to flavor the water, and feeling confident to take actions to reduce SSB consumption, such as diluting juice with water or choosing to purchase less SSB per week, were mentioned in all interviews. Feeling confident to take actions to replace SSB with water and to take actions to replace bottled water with tap water were mentioned in the majority (73%, 82%, respectively) of interviews.

Despite descriptions about acquiring skills, the majority of participants mentioned, without prompting, that their partners and other adult family and community members did not always want to change their behavior or were not supportive of their behavior change, and that this presented a potential challenge to changing the child’s beverage (Table 2, Quote 4.3). In addition, all participants mentioned that the other hands-on activities designed to see themselves as ‘role models’ (i.e., taking pictures and recording videos of themselves drinking water instead of SSB or providing water instead of SSB) or to help them decide which beverages to purchase (collection of beverage sales receipts) were difficult to complete.

*Meta-inference from mixed methods results:* After the intervention, participants felt more confident about applying the knowledge from the curriculum, but some of the designed activities were not practical. Engaging the whole family or community to support behavior change may be important.

##### Theme 5: Parental Behavior Change in Beverage Consumption

Mixed methods findings related to parental and child behavior change are displayed in Table 3.

After the intervention, parents maintained water consumption (45 oz/day) and reported a significant decrease in consumption of 100% fruit juice (−5.32 oz/day, *p* < 0.05). SSB consumption also decreased but it was not statistically significant (−3.79 oz/day, *p* = 0.26). The main contributor to SSB consumption among parents was sweetened coffee, which remained persistently high after the intervention (9–10 oz/day). The response pattern of positive behavior changes reached full consensus in the qualitative interviews (Table 3, Quote 5.1). The primary example of behavior change across all interviewees was the use of the water filter to drink tap water (Table 3, Quote 5.2). Participants also reported that they decreased the amount of added sugar in coffee or tea (Table 3, Quotes 5.4–5.5).

*Meta-inference from mixed methods results:* Although the quantitative instrument did not allow for differentiation between tap and bottled water consumption among parents, the quantitative and qualitative findings taken together suggest that it is plausible that parents started consuming filtered tap water, replacing bottled for tap to maintain their total water intake, and also reduced the amount of sugar they used to sweeten home-made drinks. This reduction was likely not captured in the quantitative surveys, which asks about overall oz. of a drink category, not recipes.

##### Theme 6: Parental Decision and Modeling in Child Beverage Consumption

Given the age of the children, child beverage consumption was decided primarily (if not entirely) by the parents. Quantitative data suggests that infant and toddler tap water consumption increased significantly by 7.04 oz/day (*p* < 0.05), while consumption of bottled water and 100% fruit juice decreased significantly (−7.52 oz/day, *p* < 0.05; −2.89 oz/day, *p* < 0.05). Composite SSB also decreased but was not statistically significant (−1.45 oz/day, *p* = 0.11). At baseline, consumption of all other SSBs was overall very low (<1 oz/day). Although the intervention strategies did not mention or target plain milk consumption, it is worth noting that consumption of plain low fat/fat free milk increased significantly (2.58 oz/day, *p* = 0.01) and there were nonsignificant changes in the consumption of 2% fat (0.22 oz/day, *p* = 0.70) and whole milk (−1.89 oz/day, *p* = 0.17) after the intervention.

During the interviews, all but one parent described child behavior change (Table 3, Quote 6.1) or explained how the intervention activities had encouraged them to feed their children more water instead of SSB (Table 3, Quotes 6.2–6.3). Although the interview guide did not prompt or ask directly about parental modeling behaviors, the theme of modeling emerge in the interviews as a potential mechanism for behavior change (Table 3, Quote 6.4).

*Meta-inference from mixed methods results:* Parents reported change in the types of beverages that children consumed. A potential mechanism is that parents themselves engaged in the desired behavior and modeled the behavior for their children.

### 2.4. Stage 4—Dissemination and Refinement of the Intervention

#### 2.4.1. Dissemination

Results from the quantitative survey evaluation were presented in graphs and other forms of data visualization to the leadership of the early child care organization and the home visitors. Illustrative quotes from the qualitative findings were also presented as potential explanations for the quantitative findings. Discussions about the interpretation of these findings ensued, taking into consideration the limitations in the study design: (1) using of the pre-post design without a control group made it impossible to state that the behavior changes measured were not influenced by child growth or external factors; (2) the self-reported dietary intake had inherent biases related to social desirability, recall, and inaccuracies in over and under-reporting of quantities; (3) the small sample size limited our ability to do meaningful stratified analyses (by infant/toddler age group, infant/toddler sex, or parents’ country of origin); (4) possible selection bias in the qualitative interviews may have limited negative perspectives about the intervention. With these limitations in mind, the leadership of the early child care organization then worked closely with the academic partner to interpret and disseminate the mixed methods findings in presentations to scientific conferences (posters), community forums (informal talks), and the writing of this manuscript, all with joined authorship.

#### 2.4.2. Refinement of Intervention

**Figure 2:**
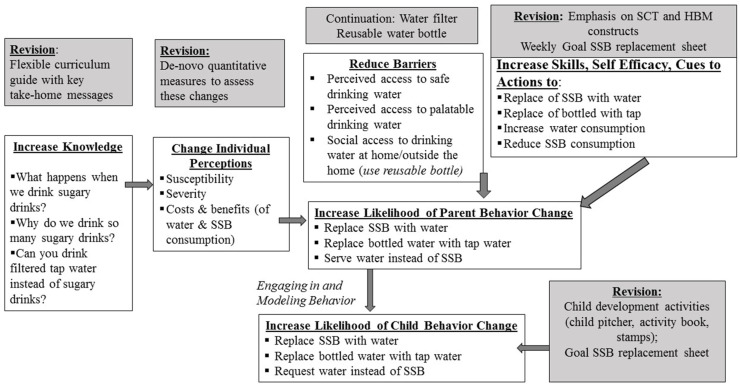
Diagram of Revised Theoretical Framework and Intervention.

Over an additional year the leadership of the early child care organization and academic partner met to incorporate the meta-inferences and feedback from the home visitors into a revised intervention. First, the theoretical framework was refined to explicitly include the following constructs in the intervention design and its evaluation: parental modeling of behaviors, parents’ perceived susceptibility to a health problem, severity of the problem, costs and benefits to behavior change, self-efficacy with respect to the desired behavior, “cues to action”, and person-environment interaction (reciprocal determinism) following a sequence based on the Health Belief Model (HBM) and Social Cognitive Theory (SCT). (Figure 2) [38,47,48]. Although the original theoretical framework implicitly included these constructs in the various domains, the revised theoretical framework made them explicit and therefore, measurable.

The intervention was revised to include activities to foster parent-child interaction and modeling behaviors that encourage reduction of SSB consumption and increase water consumption (or replacing it completely when possible). This has been documented as an important component of intervention strategies in other studies [49]. These interactive activities were co-designed to fulfill the outcomes and indicators detailed in the Early Head Start Early Learning Outcomes Framework [50]; thereby aligning better with the mission and goals of the early child care organization and providing a stand-alone curriculum. Age-appropriate materials (e.g., child water pitcher, storybooks) were added to the intervention to support the parent-child interactions.

In addition, the curriculum was re-designed as a flexible guide emphasizing key take-home messages and allowing several weeks for each ‘topic’ to be approached. The guides were organized according to three salient themes: ‘*What happens when we drink sugary drinks?*’*;* ‘*Why do we drink so many sugary drinks (awareness of marketing and availability of SSB vs water in neighborhood);* ‘*Can you drink filtered tap water instead of sugary drinks?*’. Sales receipts, video and picture-taking activities were replaced with a goal-setting worksheet that aimed to motivate parents to set a beverage goal for themselves and their children, to track their progress, and to identify barriers to progress and address those with the home visitor.

The following revisions were also made: adding one replacement filter cartridge per family; including *de-novo* survey questions to further understand the qualitative results about potential partners’, families’ and community lack of support in replacing SSB and provide insight into a future iteration of the intervention.; revising the study design to assess the importance of the curriculum plus the filter, vs. just providing a filter. In line with the CBPR approach, the academic and community partners worked together to obtain additional federal funds to test the revised intervention (ongoing trial– results not presented in this manuscript).

## 3. Discussion

This manuscript documented in detail the use of mixed methods in all CBPR phases, and explicitly described how meta-inferences were drawn from the integration of mixed methods and ensuing discussions with the community advisory board and the early child care organization. The use of a CBPMMR approach was critical to gain important insight into a dietary disparity and potential solutions in the context of this community: applying recommendations to replace SSB with plain water [51,52] without first acquiring deep understanding about how the recommendation may be adopted in communities where dietary disparities exist, may actually contribute to exacerbate these disparities. These findings are particularly important to public health fields that continue to see persistent disparities, such as the fields of nutrition, obesity and water access [53]. Specifically for SSB consumption, a risk factor for childhood obesity and diabetes, significant ethnic intake disparities have been noted at an early age [54,55,56].

The existing reviews in the field of SSB and water underscore the need to better understand how strategies may work in low-income communities and among parents of very young children [49,57,58]. CBPMMR approaches are crucial to complete this work. This manuscript contributes a step-by-step guidance on CBPMMR that can inform future interventions. Community partners’ insight about how high 100% juice consumption and its potential confusion with fruit-flavored drinks (the quantitative survey confirmed that infant and toddlers’ 100% juice consumption at baseline greatly exceeded the American Academy of Pediatrics’ guidelines [59]) increased the relevance of the intervention to the problems faced in the community. The mixed-methods formative work and leading community advisory board discussions within the context of current events uncovered the necessity of including a water filter in the intervention. We learned through the explanatory qualitative interviews that parents used less sugar to prepare home-made beverages after the intervention, with important implications to increase the sensitivity of the quantitative survey to these nuanced behavior changes.

The integration of quantitative and qualitative results often occurred in discussions with the community advisory board or with the early child care organization community partner, which led to inferences on how the intervention may have worked to achieve the desired results, and, more importantly, where the intervention design, implementation, and evaluation could be strengthened. Furthermore, the manuscript describes how CBPMMR is inevitably influenced by the convergence of current events and the community advisory board members’ lived experiences: the community advisory board’s interest in understanding tap water consumption reflected nationwide trends and current events [31,32].

## 4. Limitations

Several limitations to the partnership and evaluation must be noted. First, governance was the responsibility of the academic partnership, as a requirement of the funder; therefore, the partnership was not completely equitable. Second, the actual partnership process, stewardship, and voices in decision-making among partners were not evaluated, and would have added importantly to this analysis. CBPR best practices have been associated with outcomes and partnership sustainability [3]. Third, longitudinal evaluations of this partnership would be ideal as community engagement is a dynamic process subject to influences of the sociopolitical environment, changes in priorities, capacities, and resources of researchers and community leaders [60]. Despite these limitations, these findings raise important questions about the possibility of addressing safety of tap water as a potential approach to reduce SSB and juice consumption in this population.

## 5. Conclusions

The integration of quantitative and qualitative data in the phases of CBPR allowed for the identification of preliminary data for potential behavioral changes (increased filtered tap water and decreased bottled water consumption among children, decreased juice consumption among children and parents), insights into potential mechanisms of behavior change, and important modifications to the intervention design, implementation and evaluation to better address the nutrition needs of the community.

## Figures and Tables

**Table 1:** Characteristics of parents participating in Water Up! @Home pilot evaluation study.

Characteristics	*n* (%) or Mean (SD)
Sex *n* = 35	
Male	2 (5.71)
Female	33 (94.28)
Age categories *n* = 36	
18–25 years	5 (13.89)
26–30 years	7 (19.44)
30–34 years	13 (36.11)
35–40 years	8 (22.22)
41–65 years	3 (8.33)
Education *n* = 33	
0–6 years	15 (45.45)
7–12 years	13 (39.39)
>13 years	5 (15.15)
Country of birth *n* = 36	
El Salvador	17 (47.22)
Guatemala	12 (33.33)
Honduras	4 (11.11)
Mexico	2 (5.56)
U.S.	1 (2.78)
Number of years in US *n* = 35	
3–5 years	5 (14.28)
6–10 years	8 (22.85)
10–20 years	22 (62.86)
Household income *n* = 36	
$1–5 K	1 (2.78)
$5–10 K	3 (8.33)
$10–15 K	6 (16.67)
$15–20 K	16 (44.44)
$20–30 K	6 (16.67)
$30–40 K	3 (8.33)
$>40 K	1 (2.78)
Mean age of children in months, *n* = 36	29.53 (10.87)
Household size, *n* = 36	
1 person	1 (2.78)
2–4 people	10 (27.78)
5–6 people	14 (38.89)
>7 people	11 (30.56)
Number of hours spent with child 1 at home, *n* = 36	
4–6 h	5 (13.89)
>7 h	31 (86.11)
Where did you obtain water outside of U.S.?, *n*= 35	
Bottle	4 (11.42)
Filtered	4 (11.46)
Tap/Faucet	16 (45.71)
Well/Spring	11 (31.42)
Where did you obtain water in the U.S.?, *n* = 35	
Bottle	31 (88.57)
Filtered	2 (5.71)
Tap/Faucet	2 (5.71)
How much do you spend in water? *n* = 36	
$25 bi-weekly	8 (22.22)
$25 per week	7 (19.44)
>$25 per month	11(19.44)
$25 per month	4 (11.11)
<$25 per month	6 (13.89)

**Table 2:** STAGE 3: Integrated mixed methods results for theoretical constructs in the Water Up! @Home pilot study.

**Theme 1: Knowledge Gained after the Intervention**					
	**Survey Variable**	**Baseline, Mean (SD)**	**Follow-Up, Mean (SD)**	**Difference**	***p*-Value ^3^**
**Meta-Inference: The curriculum lessons provided participants with new knowledge about key concepts regarding health risks associated with consuming sugary drinks and benefits of drinking water.**	Knowledge Score ^1^ *n* = 27	8.07 (1.04)	9.15 (1.23)	1.07	0.002
	**Illustrative Explanatory Quotes**	1.1: “…where the water comes from, knowing the amount of sugar that we need in our bodies… and knowing how much sugar is in the sugary drinks that we consume daily”.			
		1.2: “I learned a lot because I had an… incorrect concept [about water] because I was told when I arrived in this country [the U.S.] that I could not drink tap water because it was not drinkable. Therefore, I did not trust using it. After the program was delivered, and they taught us about the water and that it was good, I understood how important it is, and how much more economical”.			
		1.3: “What I liked most was learning how much sugar we need as people. […]since we were accustomed to drinking coffee [with sugar], soda, and everything else sweet that one eats, you go over the six teaspoons”.			
		1.4: “… soda, pop, sweet coffee, drinks, it is saying that all sugar, it can give you diabetes”.			
**Theme 2: Perceptions of susceptibility, severity, and costs & benefits**					
	**Illustrative Explanatory Quotes**	2.1: “As Hispanics, we are most likely to get sick from sugar, to have diabetes, and I believe that it is because we don’t drink water, because we don’t have the information… that sugar can cause us harm in the near future if we continue drinking too many sugary drinks”.			
		2.2: “… they are things that one needs to know… because sometimes one can be causing themselves harm; speaking of diseases, primarily diabetes, which is a disease that humans can suffer from due to consuming a lot of sugar”.			
**Theme 3: Perceived physical barriers to drinking filtered tap water**					
	**Survey Variable**	**Baseline *n* (%)**	**Follow-Up *n* (%)**	***p*-Value ^3^**	
**Meta-Inference:** Concerns about tap water safety continued throughout the intervention but the use of the water filter may have mitigated some of those concerns, and motivated parents to move from bottled to tap water consumption.	**Do you drink tap water at home?** *n* = 36			0.08	
	Always	4 (11.11)	14 (38.89)		
	Sometimes	8 (22.22)	7 (19.44)		
	Never	24 (66.67)	15 (41.67)		
	**Do you give your children tap water at home?** *n* = 35			0.51	
	Always	2 (5.71)	12 (34.29)		
	Sometimes	6 (17.14)	7 (20.00)		
	Never	27 (77.14)	16 (45.71)		
	**How often do your children drink bottled water at home?** *n* = 35			0.7	
	Always	29 (82.86)	10 (28.57)		
	Sometimes	6 (17.14)	17 (48.57)		
	Never	0 (0)	8 (22.86)		
	**If your children don’t drink tap water at home, why not?** *n* = 31			0.71	
	Don’t like taste	4 (12.90)	4 (12.90)		
	Makes me sick	21 (67.74)	18 (58.06)		
	Told not to	3 (9.68)	1 (3.23)		
	Other	3 (9.68)	8 (25.81)		
	**Do you like the taste of water?** *n* = 36			0.56	
	Don’t like at all	0 (0)	1 (2.78)		
	Don’t like too much	0 (0)	0 (0)		
	Slightly Like	7 (19.44)	0 (0)		
	Like	7 (19.44)	10 (27.78)		
	Strongly Like	22 (61.11)	25 (69.44)		
	**Illustrative Explanatory Quotes**	3.1: “Tap water has lead; I say that that tap water is bad. Filtered is another thing, but I don’t like tap water”.			
		3.2: “I didn’t have a filter. I bought water and I spent a lot [of money] on water, so with this filter I save a lot”.			
		3.3: “The barrier was starting to drink water without filtering it. [After receiving the filter] everything was fine, since I was more trusting; even the children were drinking this water”.			
		3.4: “With the filter I believe that the flavor is good… I smelled the scent of the unfiltered water, and the scent of the filtered water is totally different”.			
**Theme 4: Skills and self-efficacy**					
	**Survey Variable**	**Baseline Mean (SD)**	**Follow-Up** **Mean (SD)**	**Difference**	***p*-Value^3^**
**Meta-Inference:** Intervention increased self-efficacy to drink water instead of SSB, and identified areas of future work (providing support for partners or entities outside the home for targeted behaviors).	**Self-efficacy ^2^** *n* = 28	51.57 (4.65)	54.21 (2.48)	2.64	0.007
	**Illustrative Explanatory Quotes**	4.1: “[The intervention] helped me a lot because there was a time when it was very hot… and first I thought of soda, but after I said, ‘no, I need to drink water because it is healthier’, and that is how I put into practice what I had been told”.			
		4.2: “This is the first thing I do now; before…when one does not have the adequate information one only goes and looks… to see what is on sale, but now I try to recall the [information] about the teaspoons of sugar that every drink contains and I focus on the [nutrition] label”.			
		4.3: what [was more difficult] was my husband and still today since he always says after eating: I want something sweet (,,,).he tells me always: make me a sweet tea…I want to drink something sweet’.			

^1^ Range score from 0–11. Higher scores mean higher knowledge. ^2^ Range score from 0–57. Higher scores mean higher self-efficacy. ^3^ *p*-values were obtained using chi-squared test for categorical variables and paired *t*-test for continuous variables.

**Table 3:** Integrated mixed methods results for behavioral outcomes in the Water Up! @Home pilot study.

**Theme 5: Parental behavior change in beverage consumption (*n* = 36)**				
**Survey Variable Beverage**	**Baseline Mean (SD)**	**Follow-up Mean (SD)**	**Mean Difference (95% CI)**	***p*-Value ^3^**
**Total Water Intake**	45.36	44.94	−0.42	0.91
	(14.42)	(16.11)	(−7.72, 6.88)	
**100% Fruit Juice**	8.41	3.09	−5.32	0.005
	(11.39)	(5.03)	(−8.88, −1.76)	
**Fruit-flavored drinks**	5.71	4.07	−1.64	0.19
	(8.31)	(5.28)	(−4.11, 0.84)	
**Whole Milk**	4.77	3.56	−1.21	0.35
	(6.92)	(5.27)	(−3.79, 1.36)	
**Reduced Fat Milk (2%)**	2.56	1.31	−1.25	0.19
	(6.95)	(2.81)	(−3.14, 0.64)	
**Low Fat/Fat Free Milk (1%, Skim)**	3.28	3.56	0.28	0.84
	(6.27)	(5.83)	(−2.45, 3.01)	
**Soda**	2.77	1.32	−1.45	0.11
	(5.62)	(2.77)	(−3.27, 0.36)	
**Diet Soda**	0.11	0.09	−0.02	0.86
	(0.41)	(0.35)	(−0.22, 0.18)	
**Sweet Coffee/tea**	9.71	9.29	−0.42	0.87
	(14.02)	(13.07)	(−5.49, 4.66)	
**Sports Drinks**	0.33	0.05	−0.29	0.05
	(0.85)	(0.28)	(−0.57, 0.003)	
**Composite Sugar Sweetened Beverages ^1^**	18.52	14.73	−3.79	0.26
	(19.53)	(13.02)	(−10.48, 2.89)	
***Meta-Inference*:** Participants increased filtered tap water consumption after the intervention, and likely reduced added sugar to home-made drinks, in addition to reductions reported in the quantitative survey	5.1: “Now I am no longer buying water and we don’t buy juice; we are saving a lot [of money]. I feel that it is healthier for me and my kids”.			
	5.2: “I have noticed that because of the filter… [before I bought] up to 3 boxes [of water bottles]. Now I keep only one [box] because we use [water] from the tap… the filter serves me well, because I have it here and I use it”.			
	5.3: “…for example, before I drank coffee and abundant sugar, lots of sugar I noticed…’			
	5.4: “and the coffee and what we love are the teas, I loved them with sugar, but right now I consume them but it’s not as much, I consume water first”.			
	5.5: “…now if it used to have one tablespoon, I put like one third of it”.			
**Theme 6: Parental decisions and modeling in infant and toddler beverage consumption (*n* = 36)**				
**Survey Variable Beverage**	**Baseline Mean (SD)**	**Follow-up Mean (SD)**	**Mean Difference (95% CI)**	***p*-value ^3^**
	2.04	9.08	7.04	
**Tap Water (filtered)**	(4.62)	(6.82)	(4.46, 9.61)	<0.001
**Bottled Water**	14.73			
	(6.11) 7.20			
	(6.99)			
	(7.52)			
	(−10.75, −4.29)			
	<0.001			
**100% Fruit Juice**	6.48	3.58	−2.89	0.002
	(5.13)	(3.17)	(−4.62, −1.18)	
**Fruit-flavored Drinks**	1.82	1.12	−0.69	0.18
	(3.09)	(2.03)	(−1.74, 0.34)	
**Whole Milk**	9.58	7.69	−1.89	0.17
	(8.72)	(8.23)	(−4.64, 0.86)	
**Reduced Fat Milk (2%**	1.2	1.42	0.22	0.7
	(3.3)	(3.92)	(−0.92, 1.35)	
**Low Fat/Fat Free Milk (1%, Skim)**	2.24	4.82	2.58	0.01
	(5.44)	(6.74)	(0.62, 4.54)	
**Flavored Milk**	1.19	0.29	−0.9	0.13
	(3.89)	(1.2)	(−2.09, 0.29)	
**Soda**	0.23	0.31	0.08	0.53
	(0.88)	(1.2)	(−0.19, 0.35)	
**Diet Soda**	0	0	0	NA
**Sweet Tea/Coffee**	0.31	0.44	0.12	0.4
	(1.2)	(1.45)	(−0.17, 0.42)	
**Sports Drinks**	0.02	0.18	0.16	0.2
	−0.12	−0.85	(−0.09, 0.41)	
**Caffeinated Drinks**	0	0	0	NA
**Composite Sugar Sweetened Beverages ^2^**	3.79	2.34	−1.45	0.11
	(5.82)	(3.92)	(−3.27, 0.37)	
**Meta-Inference:** Parents reported change in the types of beverages that children consume, and a potential mechanism for this change is the parents themselves engaging in the desired behavior, and modeling this behavior to their children, as well as the older children voicing their preference for the modeled behavior.	6.1: “Before, I sometimes gave my child one or two juices. Now I almost never give him juice… I give him water”.			
	6.2: “We did activities, like putting fruit in the water and drinking water together with my daughter, things like that, to encourage her to drink a little more water”.			
	6.3: “As a mom I go to the park… when the kids are playing and get thirsty, they ask for water. I carry water [with me], I don’t bring juices when I am with them”.			
	6.4: “…As a mother you eat or you feed the child, and the child looks at you and sees what you are doing, so then I also am practicing that I have to eat it so that they look at me, see me enjoying it (even if I don’t like it) […]grab my glass of water, and my youngest girl says to me: *mami*, water—because she sees me drinking water, and so then I feel happy because, really, it is something healthy for the life of my family”.			

^1^ Composite Sugar Sweetened Beverages (SSB) in parents was calculated adding the consumption of: soda, sweetened fruit beverages, sweet tea & coffee, and sports drinks.^2^ Composite SSB in children was calculated adding the consumption of: soda, sweetened fruit beverages, flavored milk, sweet tea & coffee, and sports drinks. ^3^ *p*-values were obtained from paired *t*-tests.

## Data Availability

The data presented in this study are available on request from the corresponding author. The data are not publicly available in accordance with the IRB process regarding confidentiality in participant consent procedures.
